# Phosphatase PP2A enhances MCL-1 protein half-life in multiple myeloma cells

**DOI:** 10.1038/s41419-020-03351-7

**Published:** 2021-03-03

**Authors:** Anne Slomp, Laura M. Moesbergen, Eric Eldering, Marie José Kersten, Monique C. Minnema, Victor Peperzak

**Affiliations:** 1 Center for Translational Immunology, University Medical Center Utrecht, Utrecht University, Utrecht, The Netherlands; 2 Department of Experimental Immunology, Amsterdam University Medical Centers, University of Amsterdam, Amsterdam, The Netherlands; 3 Cancer Center Amsterdam and LYMMCARE, Amsterdam, The Netherlands; 4 Department of Hematology, Amsterdam University Medical Centers, University of Amsterdam, Amsterdam, The Netherlands; 5 Department of Hematology, University Medical Center Utrecht, Utrecht University, Utrecht, The Netherlands

**Keywords:** Myeloma, Preclinical research

## Abstract

Multiple myeloma (MM), a treatable but incurable malignancy, is characterized by the growth of clonal plasma cells in protective niches in the bone marrow. MM cells depend on expression of BCL-2 family proteins, in particular MCL-1, for survival. The regulation of MCL-1 is complex and cell type-dependent. Unraveling the exact mechanism by which MCL-1 is overexpressed in MM may provide new therapeutic strategies for inhibition in malignant cells, preferably limiting side effects in healthy cells. In this study, we reveal that one cause of overexpression could be stabilization of the MCL-1 protein. We demonstrate this in a subset of MM and diffuse large B cell lymphoma (DLBCL) cell lines and MM patient samples. We applied a phosphatase siRNA screen to identify phosphatases responsible for MCL-1 stabilization in MM, and revealed PP2A as the MCL-1 stabilizing phosphatase. Using the PP2A inhibitor okadaic acid, we validated that PP2A dephosphorylates MCL-1 at Ser159 and/or Thr163, and thereby stabilizes MCL-1 in MM cells with long MCL-1 half-life, but not in DLBCL cells. Combined kinase and phosphatase inhibition experiments suggest that the MCL-1 half-life in MM is regulated by the counteracting functions of JNK and PP2A. These findings increase the understanding of the mechanisms by which MCL-1 is post-translationally regulated, which may provide novel strategies to inhibit MCL-1 in MM cells.

## Introduction

Pro-survival B cell lymphoma 2 (BCL-2) family proteins are potent inhibitors of programmed cell death and are often overexpressed in malignant cells, enabling these cells to survive in the presence of DNA damage, abnormal growth signaling, and chemotherapeutic agents^1^. MCL-1 is overexpressed in many germinal center-derived malignancies, including multiple myeloma (MM), diffuse large B cell lymphoma (DLBCL), and follicular lymphoma^2^. MCL-1 expression is essential for survival of MM^3–5^, and its overexpression in MM is associated with relapse and shorter survival^6^, making MCL-1 an attractive therapeutic target. Multiple MCL-1-specific BH3-mimetics have shown effectivity in preclinical models and are now being evaluated in clinical trials for MM and other hematologic malignancies^2,7–9^. Thus, targeting MCL-1 may be beneficial for the treatment of B cell malignancies such as MM. However, in addition to its importance for malignant cells, MCL-1 expression is important for the survival of many healthy cells and tissues, such as plasma cells^10^, B cells^11,12^, hematopoietic stem cells^13^, cardiomyocytes^14^, and neural precursor cells^15^. In order to prevent unwanted side effects of MCL-1 inhibition on healthy tissues, it is important to unravel the exact mechanism of MCL-1 upregulation in MM, because this may provide new strategies to inhibit MCL-1 in malignant cells.

MCL-1 distinguishes itself from other BCL-2 family members by its size and structure. Although the C-terminal part of MCL-1, which contains a BH3 domain that is required for its anti-apoptotic function, closely resembles other pro-survival BCL-2 family members, MCL-1 is considerably larger and has an N-terminus that is subject to extensive post-translational modification. The presence of PEST regions (enriched in proline (P), glutamic acid (E), serine (S), and threonine (T) residues), as well as the presence of four arginine pairs, allows for rapid turnover of MCL-1 (refs. ^16,17^), resulting in a half-life of 30–40 min in many cell types^18,19^. Proteasomal degradation of MCL-1 occurs upon phosphorylation^20^ and subsequent poly-ubiquitination^21^ of its PEST regions.

In its PEST regions, MCL-1 contains at least 13 putative phosphorylation sites, some of which have been experimentally studied and verified^20^. Kinases GSK-3β, ERK-1, and JNK have been shown to phosphorylate MCL-1 at Thr163, and subsequently Ser155 and Ser159, resulting in MCL-1 destabilization^20,22–25^. Multiple ubiquitin ligases have been associated with MCL-1 destabilization, including Mule, SCF^β-TrCP^, SCF^Fbw7^, APC/C^Cdc20^, and Trim17 (refs. ^21,25–29^). The deubiquitinases USP9X^30^, Ku70 (ref. ^31^), and USP13 (ref. ^32^) were shown to counteract this process and thereby stabilize MCL-1. Modification of MCL-1 by kinases and ubiquitin modifiers is highly dependent on cellular context, and the exact role and mechanism of MCL-1 stabilization in MM are unknown.

In healthy germinal center (GC) B cells, tyrosine and serine/threonine phosphatase activity was shown to be increased compared to non-GC and naïve B cells^33^. It was also observed that stability of MCL-1 is increased in GC B cells, resulting in increased protein levels, and that this increased MCL-1 stability could be reversed by treatment with okadaic acid (OA), an inhibitor targeting the PP2A phosphatase complex^34^. We hypothesize that MCL-1 overexpression in post-GC B cell malignancies is the result of increased dephosphorylation of MCL-1, which leads to stabilization of the protein. Identifying the specific phosphatase responsible for MCL-1 dephosphorylation would increase insight into MCL-1 protein regulation and may result in the identification of new treatment targets for B cell malignancies.

In this study, we show that MCL-1 is stabilized in a subset of DLBCL and MM cell lines and primary MM cells. By using a phosphatase siRNA screen, we identify PP2A as MCL-1 stabilizing phosphatase in MM, but not in DLBCL cells. Moreover, we show that in MM, PP2A activity reverses phosphorylation of MCL-1 by JNK.

## Materials and methods

### Cell culture

Cell lines L363, OPM-2, MM1.S, RPMI-8226, and SU-DHL-2 were cultured in RPMI 1640 GlutaMAX HEPES culture medium (Life Technologies) supplemented with 10% fetal bovine serum (FBS, Biowest) and 100 µg/ml penicillin–streptomycin (Gibco/Life Technologies). UM9 was cultured with 20% FBS, and NCI-H929 with 20% FBS, 1 mM sodium pyruvate (Thermo Fisher), and 50 µM β-mercaptoethanol (Life Technologies). OCI-Ly1, OCI-Ly3, OCI-Ly7, and OCI-Ly10 were cultured in Iscove’s modified Dulbecco’s medium (Life Technologies) supplemented with 20% FBS and 100 µg/ml penicillin–streptomycin. Cell lines L363, RPMI-8226, OPM-2, OCI-Ly3, OCI-Ly7, OCI-Ly1 and NCI-H929 were purchased from DSMZ, MM1.S, OCI-Ly10, and SU-DHL-2 were purchased from ATCC. Cell line UM9 was derived from an MM patient at our UMCU hospital). All cell lines were tested negative for mycoplasma before and during the experiments. Primary MM cell samples were derived from patients diagnosed at the Academic Medical Center, Amsterdam, The Netherlands. This study was conducted and approved by the AMC Medical Committee on Human Experimentation. Informed consent was obtained in accordance with the Declaration of Helsinki.

### Chemicals

In order to assess the effect of PP1 and PP2A phosphatases, cells were treated for 2 h (unless otherwise stated) with 1 μM tautomycetin (TMC) (2305, Tocris) or 100 nM OA (ALX-350-003; Enzo Life Sciences), respectively. When phosphorylation of MCL-1 was studied, all samples were treated with 20 nM bortezomib (S1013; Selleckchem) and 10 μM Q-VD-OPh (SML0063; Sigma-Aldrich). Protein half-life determination was performed using 25 μg/ml cycloheximide (CHX) (HY-12320; MedChemExpress) for 0, 1, 2, and 4 h or for 0.5, 1, and 2 h. In case of CHX experiments with OA, cells were pretreated for 2 h with OA, and OA remained present during CHX treatment. In order to determine MCL-1 half-life in primary MM samples, CD138+ cells were purified from MM bone marrow aspirates by magnetic activated cell sorting using CD138 microbeads (Miltenyi Biotec), followed by CHX treatment as described above. CDK7 inhibitor THZ1 (MedChemExpress) was used at 200 nM for 8 h in order to inhibit *MCL1* transcription. Ten micromolar of GSK-3β inhibitor CHIR99021 (Tocris Bioscience), 250 nM of MEK/ERK-1 inhibitor trametinib/GSK1120212 (Selleckchem), and 20 µM of JNK inhibitor SP600125 (Merck) were used to assess the role of kinases in MCL-1 degradation.

### Immunoblotting

For western blot analysis, cells were lysed in buffer containing 1% NP-40 and proteins were separated using SDS-PAGE (Mini-PROTEAN® TGX™ Precast Gels, Bio-Rad), transferred to low fluorescence PVDF membranes (Bio-Rad), blocked in phosphate-buffered saline (PBS) containing 2% non-fat dry milk, and stained using the following antibodies: mouse anti-α-tubulin, rabbit anti-phospho-MCL-1 (Ser159/Thr163), rabbit anti-PP2A A subunit (PPP2R1A/B; Cell Signaling), mouse anti-PP2A C subunit, alpha isoform (PPP2CA; Merck), rabbit anti-MCL-1 (Abcam), mouse anti-MCL-1 (Santa Cruz), goat anti-mouse-680RD, and goat anti-rabbit-800CW (LI-COR Biosciences). For phospho-MCL-1 immunoblotting, all PBS in washing and staining buffers was replaced with TBS. Infrared imaging was used for detection (Odyssey Sa; LI-COR Biosciences). Analysis and quantification were performed using LI-COR Image Studio and ImageJ 1.47V software.

### Quantitative PCR

Total RNA for quantitative real-time PCR was extracted using RNA-Bee (Tel-Test, Inc.) according to the manufacturer’s protocol. cDNA was produced using the RevertAid H minus first strand cDNA synthesis kit (K1632; Thermo Fisher Scientific), and qPCR was performed with SYBR Green PCR master mix (4309155; Life Technologies) using the ViiA 7 Real-Time PCR System (Thermo Fisher Scientific). The following primers were used: MCL1 forward 5′-TCGTAAGGACAAAACGGGAC-3′, MCL1 reverse 5′-CATTCCTGATGCCACCTTCT-3′, HPRT forward 5′-CCTGGCGTCGTGATTAGTGA-3′, HPRT reverse 5′-CGAGCAAGACGTTCAGTCCT-3′, UBC forward 5′-ATTTGGGTCGCGGTTCTTG-3′, UBC reverse 5′-TGCCTTGACATTCTCGATGGT-3′, YWHAZ forward 5′-ACTTTTGGTACATTGTGGCTTCAA-3′, and YWHAZ reverse 5′-CCGCCAGGACAAACCAGTAT-3′. For PPP2R2C predesigned Taqman probe sets where used (hs00902099_m1, Thermo Fisher Scientific). Results were normalized to expression of HPRT, UBC, and YWHAZ using the ΔΔCt method^35^.

### Gene expression data analysis

Using published data sets, *PPP2R2C* gene expression was assessed in different GC B cell-derived malignancies and in healthy GC B cells. The following data sets from the National Center for Biotechnology Information Gene Expression Omnibus were used: GSE2658, GSE93291, GSE93261, GSE39671, GSE87371, and GSE38697. Data sets were analyzed using the R2 Genomics Analysis and Visualization Platform.

### Phosphatase siRNA screen

A library of siRNAs against 188 human phosphatases (Dharmacon ON-TARGET*plus*® SMARTpool® siRNA Library-Human Phosphatase) was transfected into MM1.S cells using Lipofectamine RNAiMAX and 50 nM siRNA. Sixty-six hours after transfection, lysates were made for detection of MCL-1 protein levels by immunoblotting.

### Statistical analysis

MCL-1 half-life was determined using a one-phase exponential decay non-linear regression analysis model (GraphPad Prism version 8.0.1). Statistical analysis was performed using GraphPad Prism version 8.0.1. Statistical differences between experimental groups were examined by a one-way analysis of variance (ANOVA) with Sidak post hoc multiple comparison testing for Fig. 5B and a two-way ANOVA with Holm–Sidak post hoc multiple comparison testing for Fig. S3. For all tests a *p* value of <0.05 was considered statistically significant.

## Results

### MCL-1 is stabilized in post-GC B cell malignancies

**Fig. 1:**
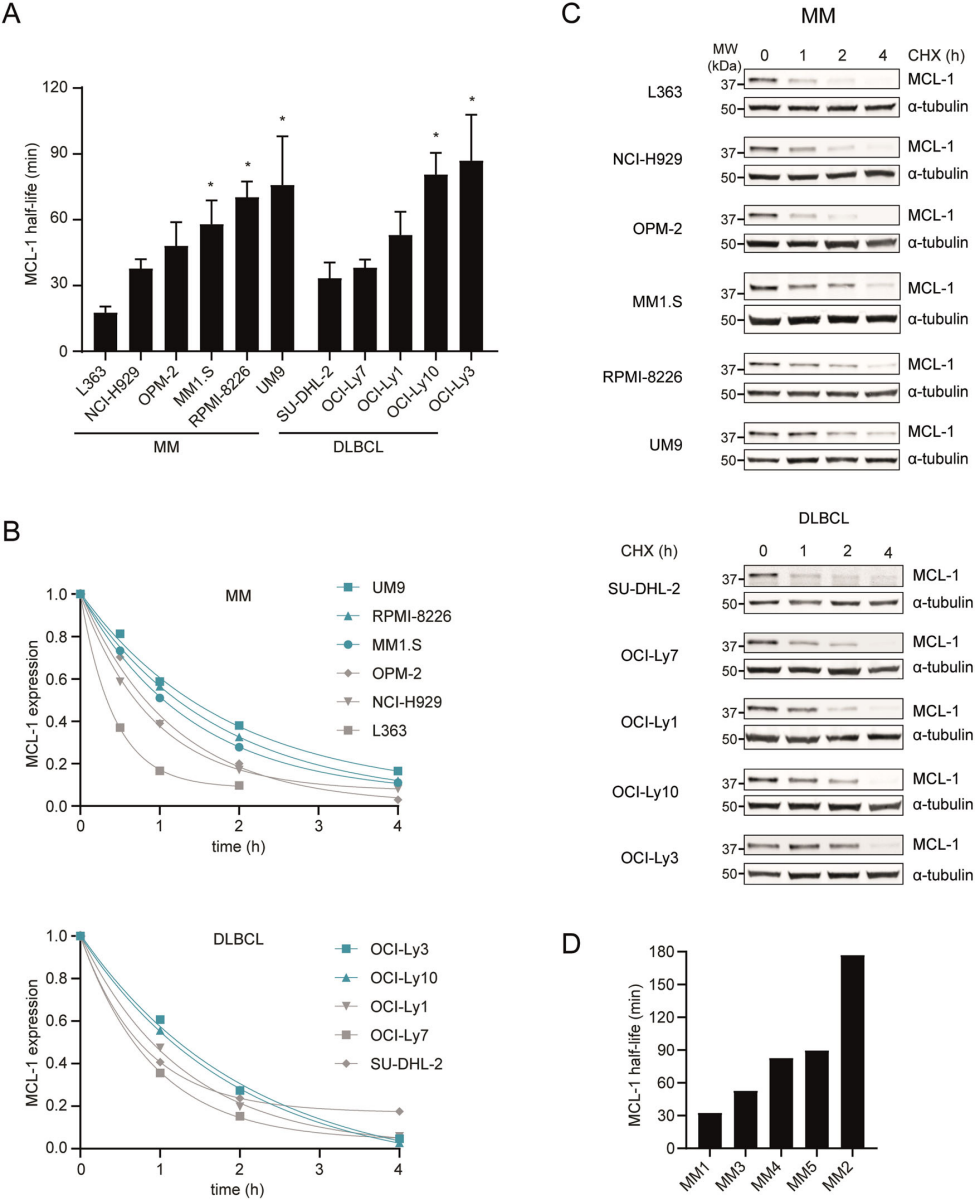
MCL-1 is stabilized in post-GC B cell malignancies. **A–C** MCL-1 protein half-life as determined by treatment of MM and DLBCL cell lines with cycloheximide (CHX). Presented data are averages of 3–6 independent replicates per cell line (*n* = 3 for RPMI-8226, L363, OCI-Ly10, and OCI-Ly7; *n* = 4 for OPM-2, OCI-Ly3, OCI-Ly1, and SU-DHL-2; *n* = 5 for MM1.S, UM9; *n* = 6 for NCI-H929). **A** Summarized data (mean + SEM) of MCL-1 half-life in 6 MM and 5 DLBCL cell lines. * indicates half-life significantly longer than 40 min, based on the 95% confidence interval. **B** Representative immunoblot images showing MCL-1 levels after CHX treatment for the indicated time periods. **C** Non-linear regression analysis (one-phase decay) of MCL-1 degradation over time after CHX treatment. **D** MCL-1 half-life in primary (CD138-purified) cells from 5 MM bone marrow aspirates, analyzed by non-linear regression analysis.

The MCL-1 protein half-life is reported to be 30–40 min in many cell types and tissues^18,19^. By treating cell lines with CHX for different time periods, we determined the half-life of MCL-1 in a panel of MM and DLBCL cell lines (Fig. 1A–C). In some cell lines, such as MM cell lines L363, NCI-H929, OPM-2, and DLBCL cell lines OCI-Ly7, OCI-Ly1, and SU-DHL-2, the MCL-1 half-life is relatively short and in line with published half-lives. In contrast, MM cell lines MM1.S, RPMI-8226, and UM9, as well as DLBCL cell lines OCI-Ly10 and OCI-Ly3 are characterized by more stable MCL-1, with a half-life significantly longer than 40 min. In order to determine whether stabilization of MCL-1 is also observed in primary MM cells, the half-life of MCL-1 was determined in CD138-purified cells from MM bone marrow aspirates (Fig. 1D). In five tested patient samples, the MCL-1 half-life ranged from 32 to 177 min.

### MCL-1 protein level reflects transcriptional activity as well as protein half-life

**Fig. 2:**
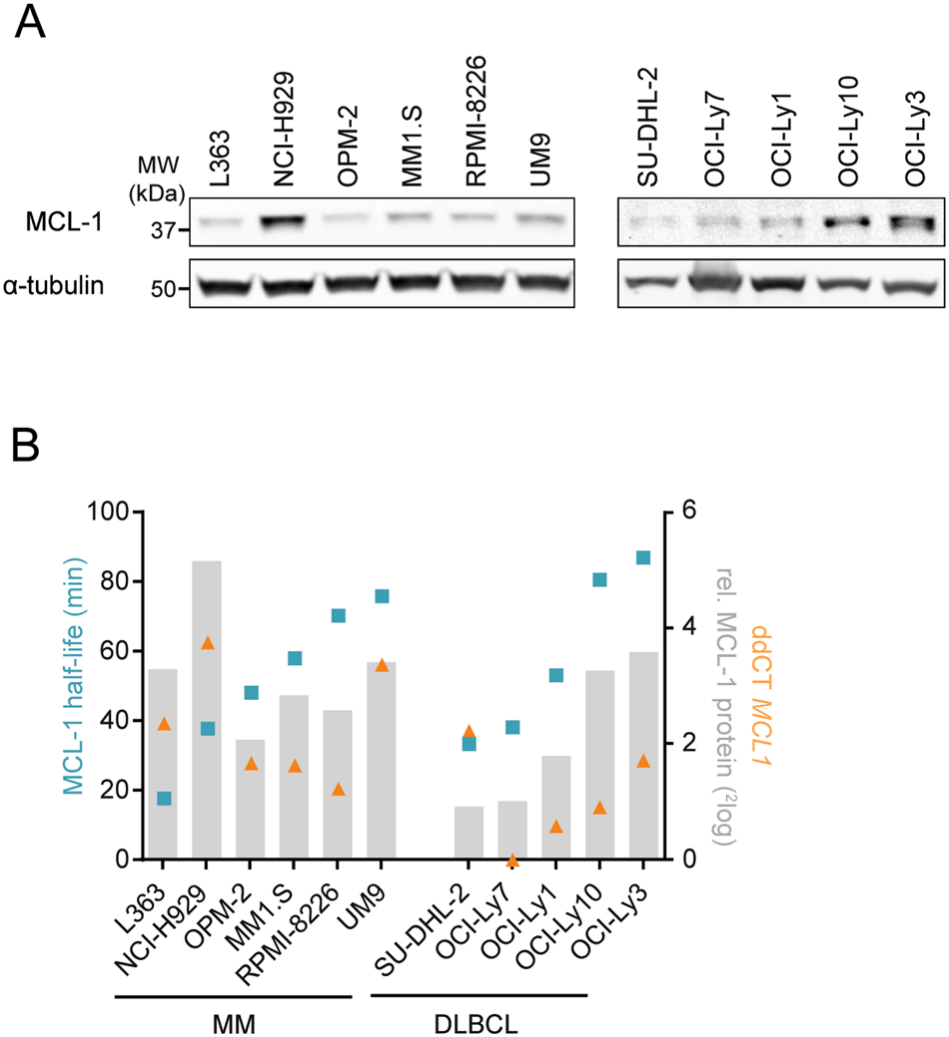
MCL-1 protein level reflects transcriptional activity as well as protein half-life. **A** MCL-1 protein expression in the MM and DLBCL cell line panel. **B** Relative MCL-1 protein expression (over α-tubulin) in the MM and DLBCL cell line panel as determined by immunoblotting (gray bars, right *y*-axis), compared to MCL-1 protein half-life (blue squares, left *y*-axis) and *MCL1* gene expression as determined by RT-PCR (orange triangles, right *y*-axis). MCL-1 gene and protein expression values are averages of two experiments. MCL-1 half-lives are averages of 3–6 independent replicates per cell line as outlined per cell line for Fig. 1A.

In order to investigate whether increased MCL-1 stability is mirrored by an increased amount of protein, we determined MCL-1 protein expression across the MM and DLBCL cell line panel (Fig. 2A). In the DLBCL cell lines, the amount of MCL-1 protein seems to correlate well with protein stability (Fig. 2B). This was not the case in the MM cell lines, where high MCL-1 protein levels were observed in NCI-H929, while this cell line has a normal MCL-1 half-life. If high MCL-1 protein levels in NCI-H929 are not caused by protein stabilization, they are likely to result from high *MCL1* transcription. To address this, the rate of *MCL1* transcription was determined by reverse transcriptase PCR (RT-PCR) analysis for each cell line, and both the rate of transcription and protein half-life of MCL-1 were compared to the total MCL1 protein level (Fig. 2B). This analysis reveals that high MCL-1 protein expression in MM cells can be a consequence of high gene transcription, increased protein half-life, or both. In contrast to MM, MCL-1 protein expression in DLBCL cells seems to be mainly correlated to protein half-life.

### Phosphatase siRNA screen identifies PP2A as MCL-1-stabilizing phosphatase complex

**Fig. 3:**
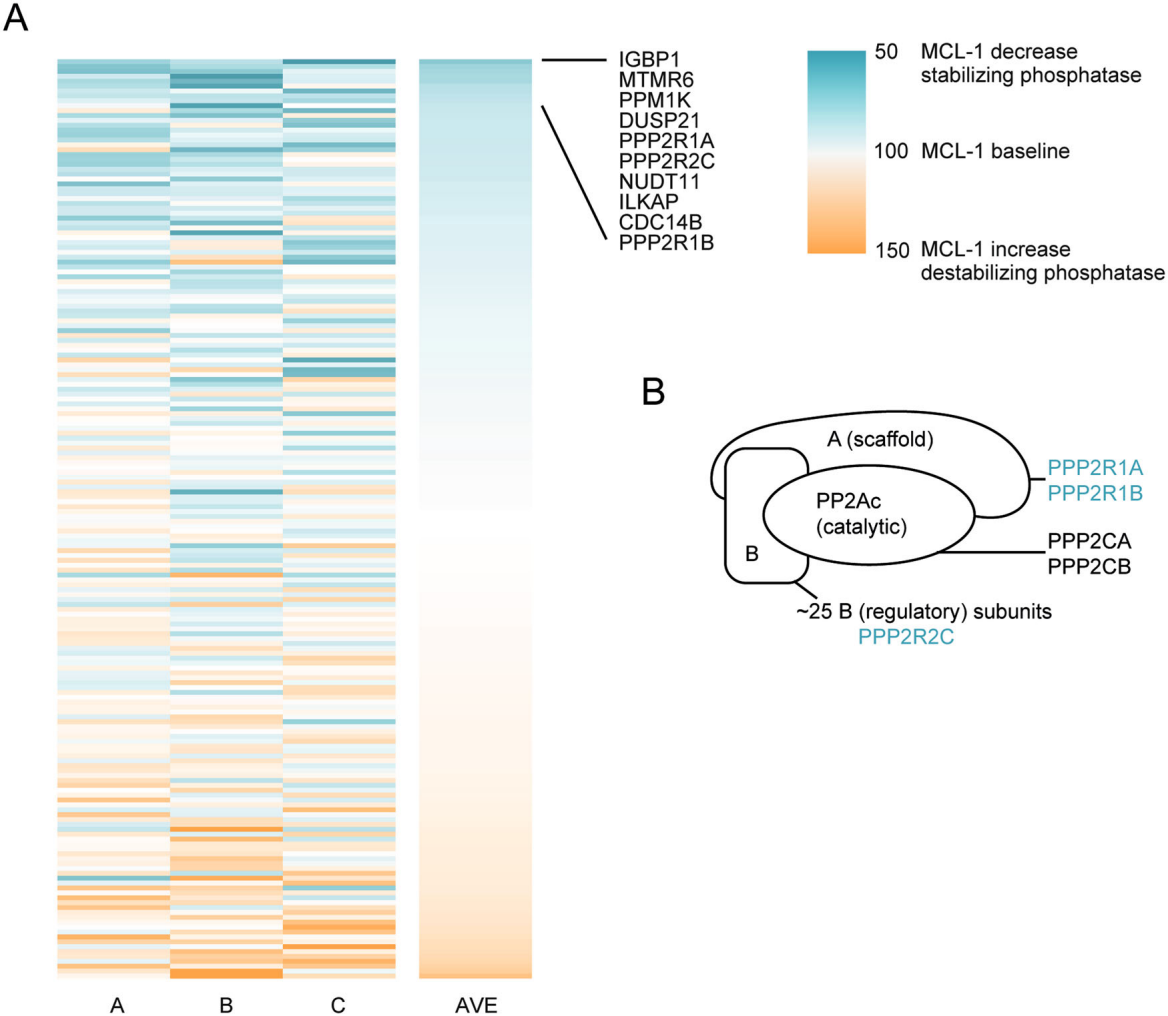
Phosphatase siRNA screen identifies PP2A as MCL-1 stabilizing phosphatase. **A** Heatmap indicates changes in the MCL-1 level after phosphatase knockdown (*t* = 66 h). The phosphatase siRNA screen was performed in three independent replicates (named A, B, or C), after which the 188 included siRNA pools were ranked based on average MCL-1 expression (AVE). The top 10 hits are named in the figure. **B** Schematic presentation of the three subunits of PP2A, with subunits that were identified in the phosphatase screen shown in blue.

Phosphatases stabilizing MCL-1 may be interesting therapeutic targets because their inhibition is expected to promote proteasomal degradation of MCL-1. Since the function of reported MCL-1-modifying proteins is highly cell type-specific, and no specific phosphatases have yet been linked to MCL-1 stabilization in MM, an unbiased approach was taken to identify phosphatases that dephosphorylate MCL-1 in MM. To this end, an siRNA screen targeting 188 phosphatases and phosphatase subunits was performed in MM cell line MM1.S (Fig. 3A). The screen was performed in three independent experiments, using altered MCL-1 protein expression as readout.

The phosphatase screen contained siRNAs against 188 phosphatases, 117 (62%) of which were protein phosphatases (Fig. S1A). The top 10 hits identified in the screen (Fig. 3A) contained 7 protein phosphatases, of which 5 had Ser/Thr and 2 had dual phosphatase specificity (Fig. S1B). MCL-1 contains 13 reported putative phosphorylation sites in the PEST regions of the protein (Fig. S1C)^20,36^. Since 12 of these sites are Ser or Thr residues, the phosphatase that dephosphorylates MCL-1 is most likely to be of Ser/Thr or dual specificity. Among the top 10 phosphatases whose knockdown diminished MCL-1 protein levels were three subunits of phosphatase complex PP2A (PPP2R1A, PPP2R2C, and PPP2R1B), and a PP2A-interacting protein (IGBP1; ref. ^37^). Therefore, PP2A was identified as potential MCL-1-stabilizing phosphatase. PP2A is a Ser/Thr phosphatase complex consisting of three subunits: a catalytic, scaffold, and a regulatory subunit (Fig. 3B). Substrate specificity and affinity of PP2A is determined by the specific regulatory subunit present in the complex^38^. In the siRNA screen, both scaffold subunits PPP2R1A and PPP2R1B were identified, as well as one regulatory subunit (PPP2R2C).

### PP2A prevents MCL-1 degradation in MM with long MCL-1 half-life, but not in DLBCL

**Fig. 4:**
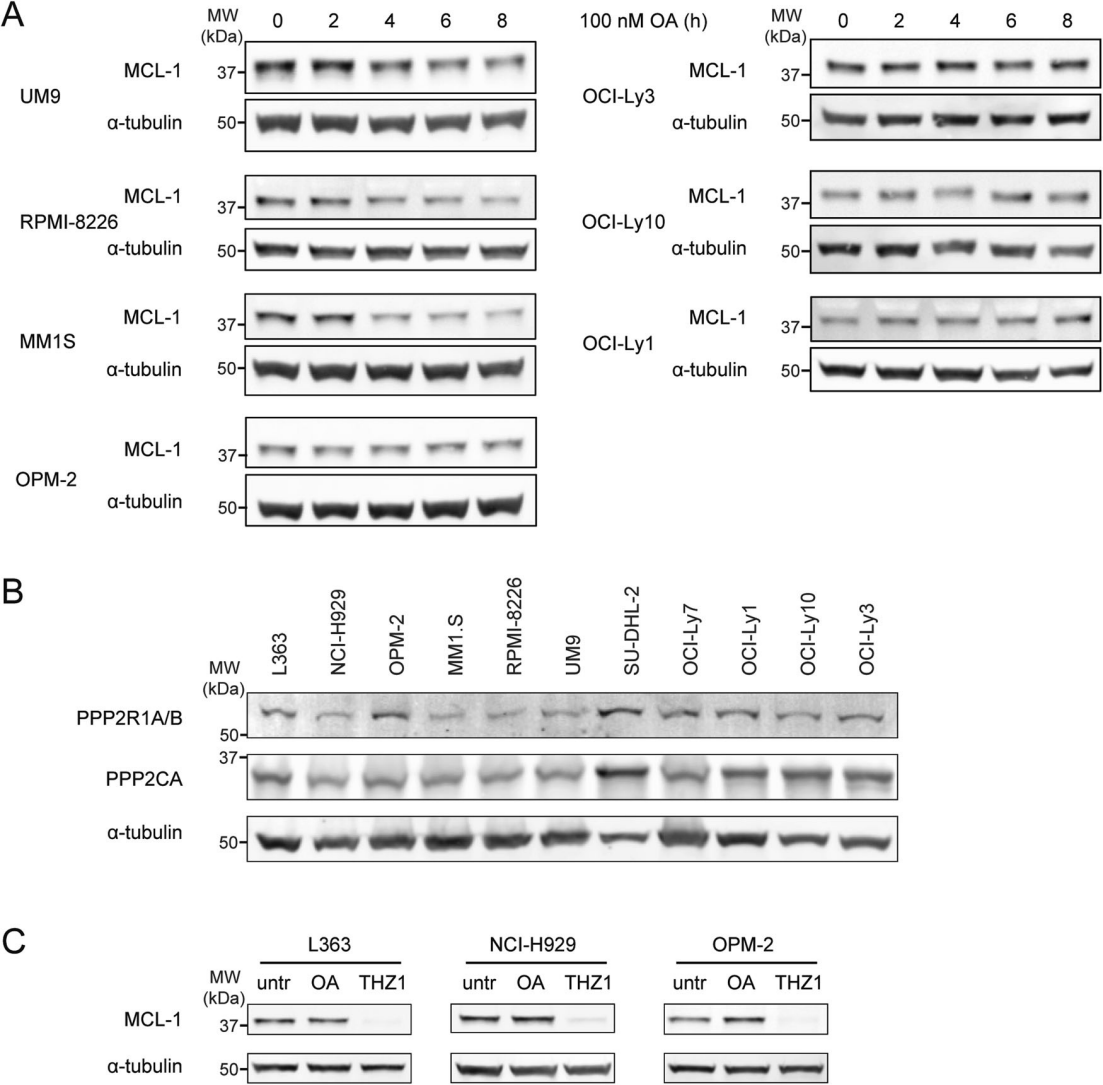
PP2A prevents MCL-1 degradation in MM with long MCL-1 half-life, but not in DLBCL. **A** MCL-1 protein levels after treatment with 100 nM PP2A inhibitor okadaic acid (OA) for the indicated time periods in MM cell lines (left) and DLBCL cell lines (right), in the presence of 10 µM pan-caspase inhibitor Q-VD-OPh. **B** Expression of the PP2A catalytic subunit PPP2CA and scaffold subunits PPP2R1A/B in the MM and DLBCL cell line panel. **C** MCL-1 protein levels after 8 h treatment with 100 nM of OA or 200 nM of CDK7 inhibitor THZ1 in MM cell lines with short MCL-1 half-life.

To explore PP2A as potential MCL-1-stabilizing phosphatase, this phosphatase complex was further studied using OA, a small-molecule PP2A inhibitor. Treatment of MM and DLBCL cell lines with OA resulted in a reduction of MCL-1 in MM cell lines where MCL-1 has a long half-life, but not in cell lines where MCL-1 has a short half-life (Fig. 4A). Moreover, the reduction in MCL-1 was observed in none of the tested DLBCL cell lines, regardless of MCL-1 half-life, suggesting that MCL-1 stabilization in DLBCL may result from the action of other phosphatases or another mechanism altogether.

Immunoblot analysis revealed that the PP2A catalytic (C) subunit PPP2CA and scaffold (A) subunits PPP2R1A and PPP2R1B are present in all tested MM and DLBCL cell lines (Fig. 4B). Although no functional antibody was available to measure expression of regulatory subunit PPP2R2C by immunoblot, we did observe mRNA expression in MM cell lines with stabilized MCL-1 and in primary MM samples (Fig. S2). In fact, analysis of published data sets revealed that PPP2R2C gene expression was significantly higher in primary MM compared to healthy GC B cells and higher compared to other GC B cell-derived malignancies (Fig. S3)

Due to the absence of MCL-1 degradation upon OA treatment in DLBCL, the effect of OA on MCL-1 was further studied only in MM.

RT-PCR analysis of OA-treated MM cell lines indicates that OA does not decrease transcriptional levels of *MCL1* (Fig. S4), suggesting that PP2A regulates MCL-1 protein stabilization rather than transcriptional regulation. In MM cell lines with short MCL-1 half-life, which are expected to rely more on *MCL1* transcription than on protein stabilization, no reduction in MCL-1 was seen following OA treatment (Fig. 4A, C). To further show that MCL-1 protein expression in these cell lines is determined by gene transcription we tested the impact of an inhibitor of *MCL-1* transcription. Cyclin-dependent kinase (CDK) 7 inhibition by THZ1 was previously shown to inhibit transcription of *MCL1*, among other genes^39^. As expected, treatment with THZ1 strongly reduced MCL-1 protein levels in L363, NCI-H929, and OPM-2 cells (Fig. 4C).

### PP2A inhibition leads to phosphorylation and destabilization of MCL-1

**Fig. 5:**
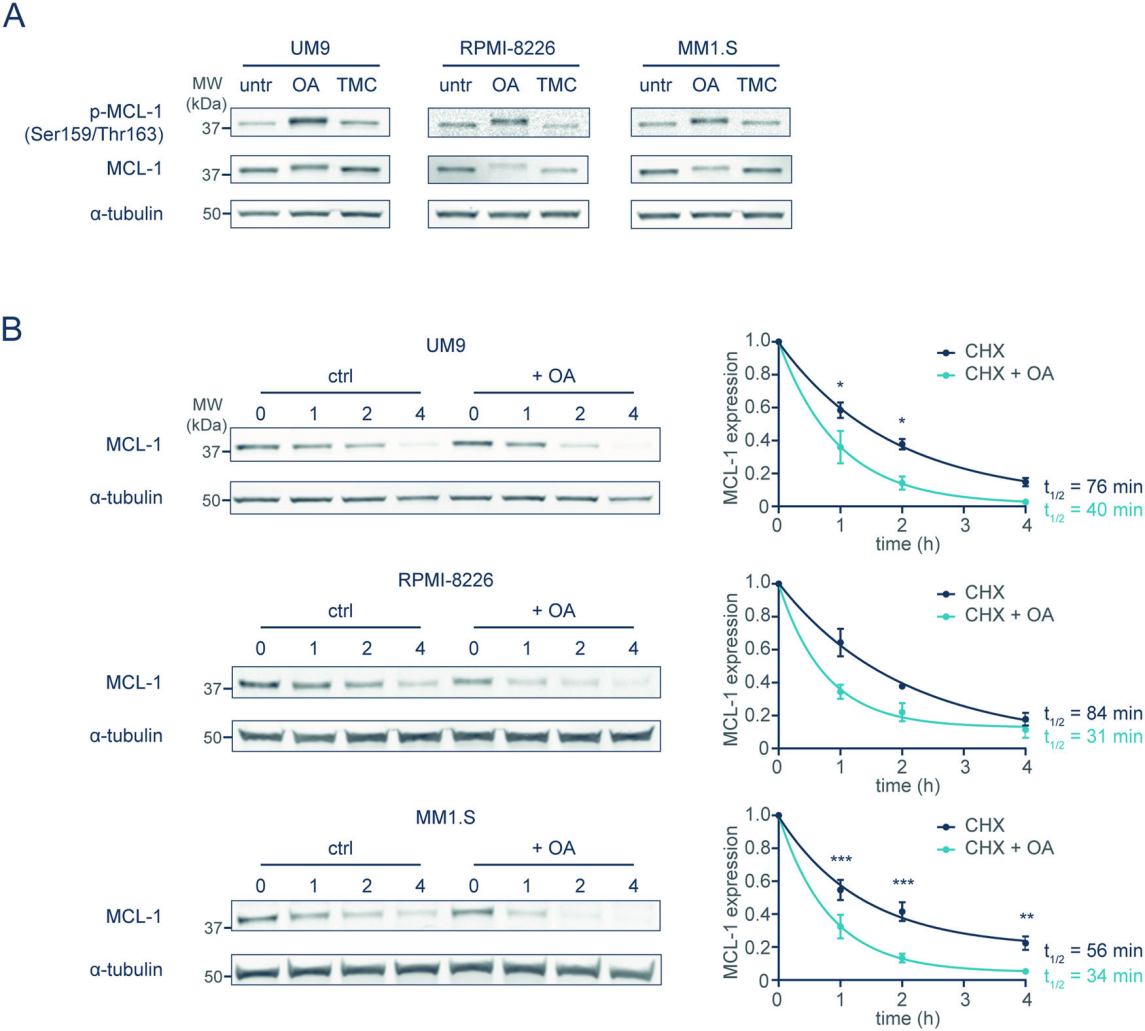
PP2A inhibition leads to phosphorylation and destabilization of MCL-1. **A** Phosphorylation of MCL-1 (residues Ser159/Thr163) after treatment of MM cell lines with long MCL-1 half-life for 2 h with either 100 nM OA or 1 µM PP1 inhibitor tautomycetin (TMC). All samples were treated in the presence of 20 nM proteasome inhibitor bortezomib and 10 μM Q-VD-OPh in order to prevent proteasomal degradation of phosphorylated MCL-1, while maintaining cell viability. **B** MCL-1 half-life after treatment with OA. Experiments and analyses were performed as in Fig. 1A–C. OA-treated samples received 2 h pretreatment with OA, and OA remained present during CHX treatment. Graphs show averages of three independent replicates ±SEM.

MCL-1 degradation is thought to be preceded by phosphorylation, which may be reversed by PP2A. In concordance with this hypothesis, OA treatment increased MCL-1 phosphorylation at Ser159/Thr163 in MM cell lines with stabilized MCL-1, when the final step of MCL-1 degradation was prevented using a proteasome inhibitor. No enhanced MCL-1 phosphorylation was observed after treatment with TMC, a PP1 inhibitor (Fig. 5A).

Since these results indicate that PP2A dephosphorylates MCL-1, leading to increased MCL-1 protein levels, it is expected that OA treatment shortens MCL-1 half-life. Indeed, CHX experiments reveal that OA reduces MCL-1 half-life, confirming that PP2A activity leads to increased MCL-1 stability in a subset of MM (Fig. 5B).

### JNK inhibition rescues MCL-1 destabilization after OA treatment

**Fig. 6:**
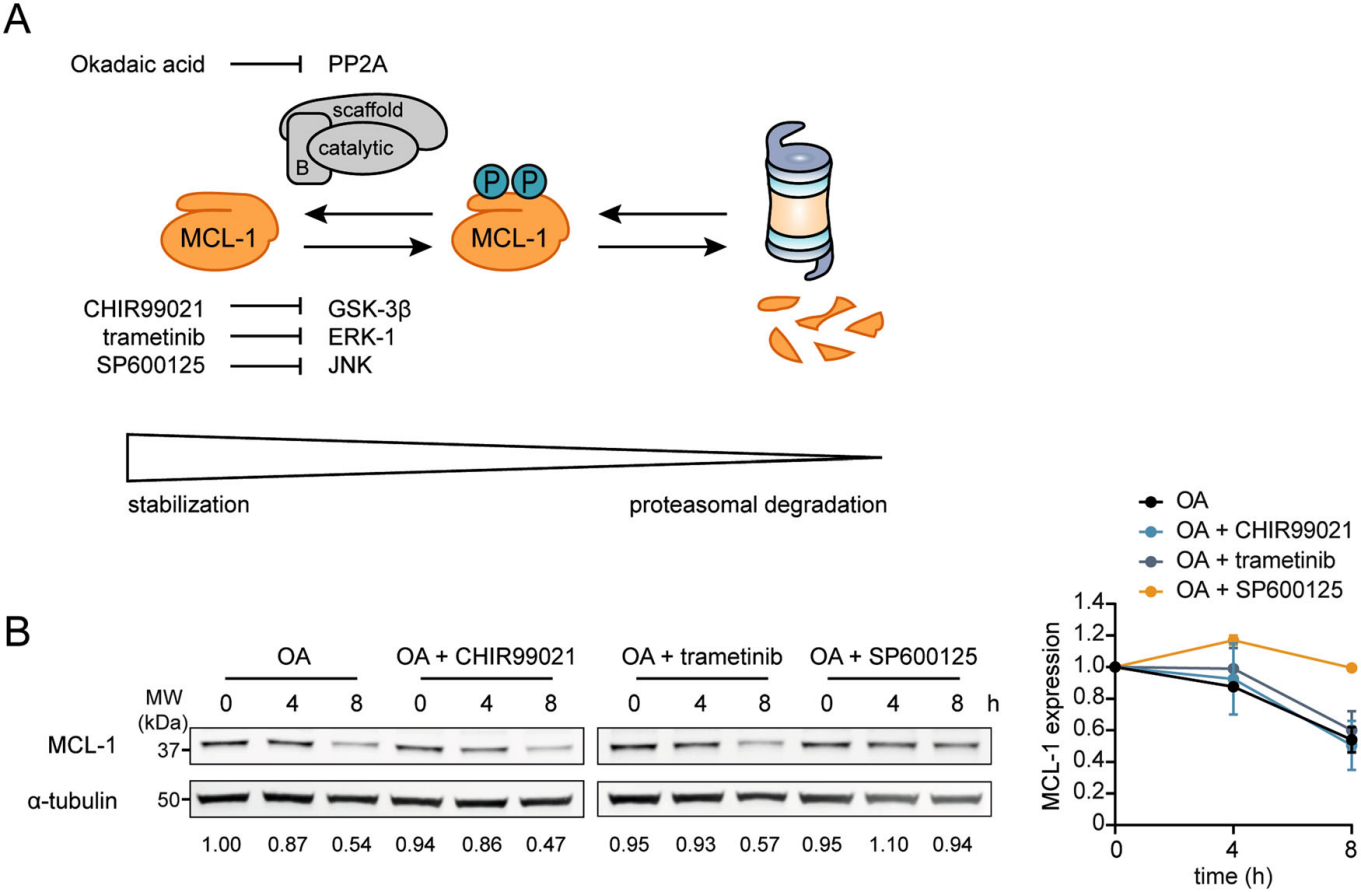
PP2A reverses MCL-1 phosphorylation by JNK. **A** Model of MCL-1 phosphorylation leading to proteasomal degradation, which is counteracted in a subset of MM by PP2A phosphatase activity. Potential kinases phosphorylating and destabilizing MCL-1 are GSK-3β, ERK-1, and JNK. The PP2A and kinase inhibitors used in this study are indicated. **B** MCL-1 expression in MM1.S after treatment for 0, 4, and 8 h with 100 nM OA, either alone or combined with 10 µM CHIR99021, 250 nM trametinib, or 20 µM SP600125. Data represent two independent experiments. Quantified MCL-1 levels (normalized to α-tubulin level and relative to untreated) are shown below the bands and are average of two experiments. The graph shows averages of two independent replicates ±SEM.

Phosphorylation of MCL-1 on Ser155, Ser159, and Thr163 was previously reported to target MCL-1 for degradation. Our results indicating that PP2A inhibition leads to increased phosphorylation at Ser159/Thr163 (Fig. 5A) suggest that PP2A can counteract kinase activity at these particular phosphorylation sites. Kinases that have been shown to phosphorylate MCL-1 at Ser155, Ser159, and Thr163 are GSK-3β, ERK-1, and JNK. In order to investigate whether one or more of these kinases is responsible for MCL-1 phosphorylation that is subsequently counteracted by PP2A, we combined OA treatment of MM1.S cells with specific kinase inhibition (Fig. 6A). GSK-3β, ERK-1, and JNK were inhibited using CHIR99021, trametinib, or SP600125, respectively. Of these inhibitors, only JNK inhibitor SP600125 was able to rescue MCL-1 stability during OA treatment (Fig. 6B). Hence, PP2A stabilizes MCL-1 by counteracting phosphorylation by JNK, but not by GSK-3β or ERK-1.

## Discussion

MCL-1 protein expression in MM cells is regulated at multiple levels. Firstly, overexpression can occur at the transcriptional level, resulting from signals from the bone marrow microenvironment or from amplification of 1q21, the locus that contains the *MCL1* gene^2,40^. 1q21 amplification occurs in ~30% of newly diagnosed and ~70% of relapsed MM patients, and is associated with increased *MCL1* expression as well as enhanced MCL-1 inhibitor sensitivity^41,42^. Secondly, alternative splicing and mRNA-regulation can influence the amount of MCL-1 protein translation^43,44^. Thirdly, MCL-1 is subject to heavy post-translational regulation, which determines its stability and function^20^.

Some phosphorylation sites of MCL-1 have been experimentally studied in cell types other than MM. In general, phosphorylation of MCL-1 at Thr163 is considered instrumental for regulation of protein stability and function, since this modification appears to be necessary for phosphorylation of other residues^23^. In conjunction with phosphorylation at Thr163, residues Thr92, Ser121, Ser155, and Ser159 can be phosphorylated. Phosphorylation of these sites has been associated with stabilization or destabilization of MCL-1 (refs. ^19,22–25,45^). Based on our observation that PP2A dephosphorylates MCL-1 at Ser159/Thr163 (Fig. 5A), which was also shown by others in lymphoma cells^46^, we studied the kinases that can phosphorylate MCL-1 at these residues: GSK-3, ERK-1, and JNK. Of these kinases, only JNK inhibition was able to rescue MCL-1 stability upon OA treatment in MM (Fig. 6). Therefore, we conclude that JNK and PP2A together regulate MCL-1 protein stability in MM.

In contrast to the extensively studied MCL-1-modifying kinases, not much is known about the mechanisms of MCL-1 dephosphorylation. Classically, phosphatases are considered promiscuous and therefore less relevant to study in the context of specific cellular pathways than kinases. The genome encodes approximately threefold less phosphatases than kinases^47,48^, and phosphatases were thought to have a non-specific function in phospho-protein homeostasis^49^. More recent findings, however, have shown that phosphatases play an active role in regulating cellular levels of tyrosine, threonine, and serine phosphorylation. Serine/threonine phosphatases such as PP2A act as holoenzymes containing a catalytic subunit, sometimes a scaffold subunit, and a regulatory subunit that recruits substrates to the complex^38,50^. As a result, PP2A can have many specific targets, depending on the regulation and availability of its regulatory subunits^50^.

We identified PP2A as the phosphatase that stabilizes MCL-1 in a subset of MM. In DLBCL, MCL-1 stabilization was observed in a subset of cell lines, but this did not result from PP2A activity. Immunoblotting indicated that the catalytic and scaffold subunits of PP2A are expressed in all tested MM and DLBCL cell lines (Fig. 4B). It is possible that the regulatory PP2A subunit responsible for MCL-1 targeting is lacking in DLBCL cells. The phosphatase siRNA screen identified one regulatory PP2A subunit, PPP2R2C. Unfortunately, we were unable to stain this subunit in immunoblot experiments, and we can therefore not validate whether PPP2R2C was a true hit from the screen responsible for MCL-1-specificity of PP2A. Regardless, MCL-1 stabilization in DLBCL likely results from a PP2A-independent mechanism, possibly by other phosphatases, by kinases, or at the ubiquitin level.

We observed increased MCL-1 stability in three out of six MM cell lines and in four out of five tested primary MM samples MCL-1 half-life exceeded the published 30–40 min^18,19^. In two cell lines with the shortest MCL-1 half-life, L363 and NCI-H929, the high MCL-1 protein levels likely result from high transcription of *MCL1*. It is conceivable that different processes contribute to high MCL-1 protein levels in MM patients as well. Thus, besides 1q21-amplified MM, where *MCL1* transcription is increased^41^, there may also be MM patients with increased MCL-1 stability, as can be expected based on our findings with primary MM cells ex vivo.

In addition to PP2A subunits, IGBP1 (Immunoglobulin-Binding Protein 1, alternatively named Alpha 4), was identified in the screen. IGBP1 does not have intrinsic phosphatase activity but is a regulator of PP2A activity. IGBP1 binds catalytic PP2A subunits and protects them from proteasomal degradation, and removal of IGBP1 was shown to result in loss of PP2A expression, leading to apoptosis^37,51,52^. The observation that IGBP1 knockdown in our screen consistently led to reduced MCL-1 levels therefore supports the hypothesis that PP2A stabilizes MCL-1.

Despite PP2A often being considered a tumor suppressor that is downregulated or mutated in cancer^53–56^, PP2A activity is essential for cell survival and its inhibition is thus a promising anti-cancer strategy^57^. This paradoxical role of PP2A in cancer likely results from the plethora of different cellular functions in which PP2A is involved, depending on the exact composition of catalytic, scaffold, and regulatory domains. Although the development of specific phosphatase inhibitors lags behind kinase inhibitor development, increasing knowledge about phosphatase biology shows that it is possible to generate small-molecule phosphatase inhibitors^58^. In a phase 1 clinical trial, the PP2A inhibitor LB100 was deemed safe to continue further development^59^. LB100 has strong chemo- and radiosensitizing potential in cancer^57,60^, indicating that PP2A inhibition could be a promising therapeutic strategy. LB100 was shown to overcome cellular senescence and promote mitotic catastrophe and apoptosis^57^. Because of the chemosensitizing potential of LB100, it would be highly relevant to address the interplay between PP2A inhibitors and established MM drugs. As an alternative to systemic inhibition of all PP2A, generation of a PP2A inhibitor that only inhibits the specific MCL-1-targeting regulatory subunit would be a promising future option.

In conclusion, we show that MCL-1 is stabilized in a subset of MM and DLBCL, and that PP2A is the phosphatase responsible for MCL-1 dephosphorylation and stabilization in MM. This finding increases the understanding of post-translational regulation of MCL-1, which may provide novel strategies to inhibit MCL-1 in MM cells.

## Supplementary information

Supplementary figure legends

Supplementary Figure 1

Supplementary Figure 2

Supplementary Figure 3

Supplementary Figure 4
