# Supplementary material for: Phosphatase PP2A enhances MCL-1 protein half-life in multiple myeloma cells
Source: Cell Death Dis. 2021 Mar 3;12(3):229. doi: 10.1038/s41419-020-03351-7 (PMC7930201; doi:10.1038/s41419-020-03351-7)
Supplement: Supplementary file 1 — Supplementary figure legends [file 41419_2020_3351_MOESM1_ESM.docx]

Supplementary Figure legends

**Figure S1.** A phosphatase siRNA screen was used to determine which phosphatases stabilize MCL-1. (**A**) Pie-charts indicating the composition of the phosphatase siRNA screen that was used to identify MCL-1-stabilizing phosphatases. The majority (117/188) of siRNA pools included in the screen targeted protein phosphatases (left), of which Ser/Thr, Tyr, and dual specificity phosphatases were all represented (middle). Among the 52 targeted Ser/Thr phosphatases and phosphatase subunits, half were PP1 and PP2A subunits (right), indicated as “PPP1” and “PPP2A” in the graph. (**B**) Types of phosphatases identified as top 10 MCL-1-stabilizing phosphatases from the phosphatase siRNA screen, and the reduction in MCL-1 level that results from knockdown of these phosphatases, relative to an siRNA pool that targets MCL-1 (siMCL1). (**C**) Schematic representation of MCL-1, its domains, and its predicted phosphorylation sites, some of which have been experimentally confirmed. The scale below indicates the size of MCL-1 in amino acids.

**Figure S2.** MM cells express PPP2R2C mRNA. (**A**) A gBlock containing the full *PPP2R2C* gene sequence was used as template for a quantitative RT-PCR and titrated in 10-fold dilution steps. This indicates the specificity of the RT-PCR. Data points are averages of 2 independent experiments. (**B**) Relative *PPP2R2C* gene expression levels as determined by RT-PCR in MM cell lines (black bars), DLBCL cell lines (dark grey bars) and primary MM patient samples (light grey bars, samples contain >85% plasma cells). *PPP2R2C* gene expression was controlled for expression of household gene *HPRT* and relative to MM cell line H929 that was taken along in every experiment. Bars represent the average of 2-4 independent experiments (*n* = 2 for MM1.s, OCI-Ly7, pMM1, pMM2 and pMM3; *n* = 3 for L363, RPMI-8226, OCI-Ly1, OCI-Ly10 and OCI-Ly3; *n* = 4 for NCI-H929, OPM-2 and SU-HDL-2) (+ S.E.M.).

**Figure S3.** PPP2R2C is expressed in primary MM cells and other post-GC malignancies. *PPP2R2C* gene expression in published Affymetrix microarray datasets from patients with indicated post-GC malignancies (MM: GSE2658, n = 542; MCL: GSE93291, n = 122; FL: GSE93261, n = 149; CLL: GSE39671, n = 130; DLBCL: GSE87371, n = 223) compared to expression in healthy GC B cells (GC: GSE38697, n = 8). Bars represent the averages of data values in each group (+ S.E.M.) and statistical analysis was relative to GC with *P* < 0.01*, *P* < 0.001** and *P* < 0.0001***.

**Figure S4.** Okadaic acid treatment does not reduce *MCL1* transcription in MM cell lines with short or long MCL-1 half-life. Relative *MCL1* gene expression levels as determined by RT-PCR, after treatment for 4 or 8 hours with 100 nM OA, in MM cell lines with long (blue bars) or short (grey bars) MCL-1 protein half-life. Bars represent the average of 3 independent experiments (+ S.E.M.).
