# Supplementary figures and images for: Phosphatase PP2A enhances MCL-1 protein half-life in multiple myeloma cells
Source: Cell Death Dis. 2021 Mar 3;12(3):229. doi: 10.1038/s41419-020-03351-7 (PMC7930201; doi:10.1038/s41419-020-03351-7)

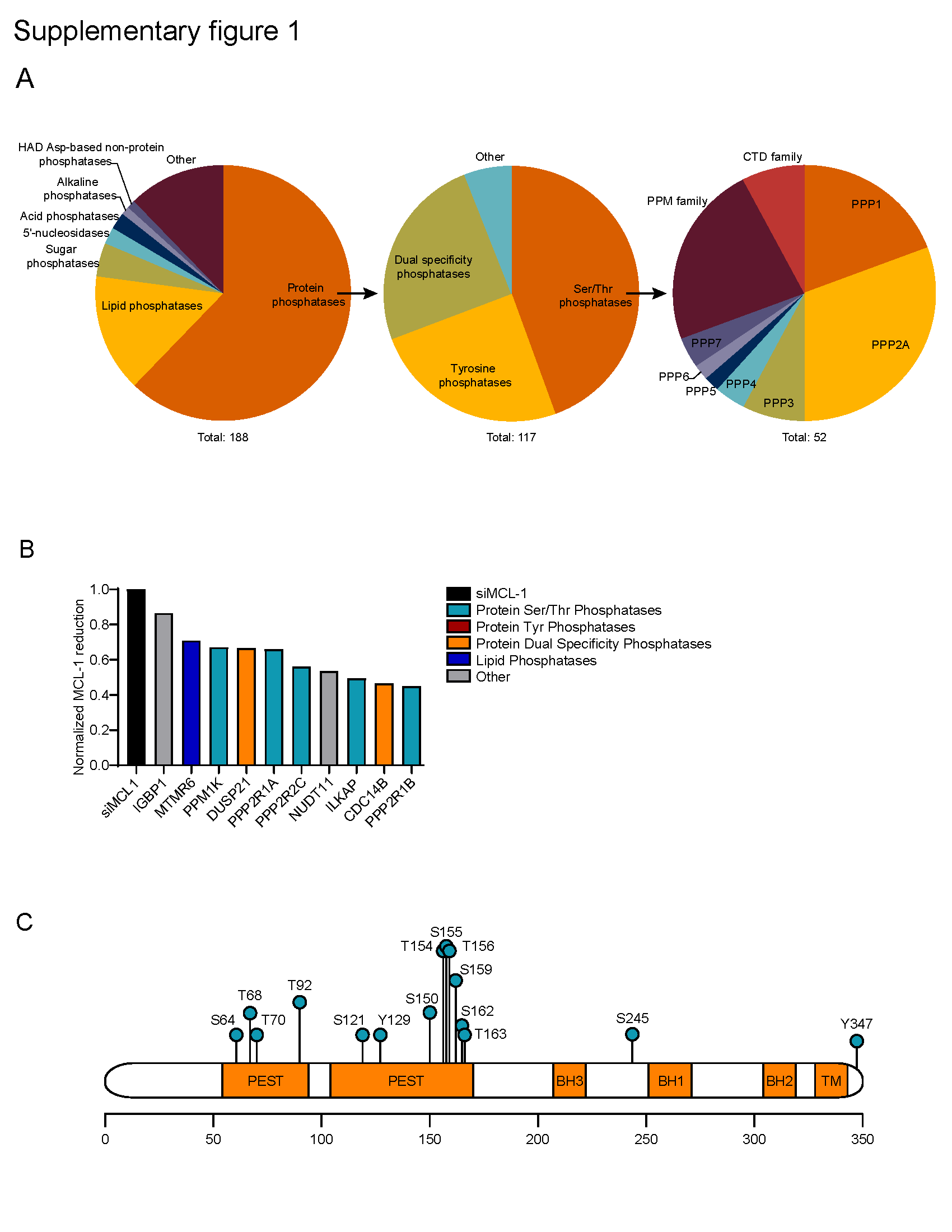

Supplement: Supplementary file 2 — Supplementary Figure 1 [file 41419_2020_3351_MOESM2_ESM.tif]

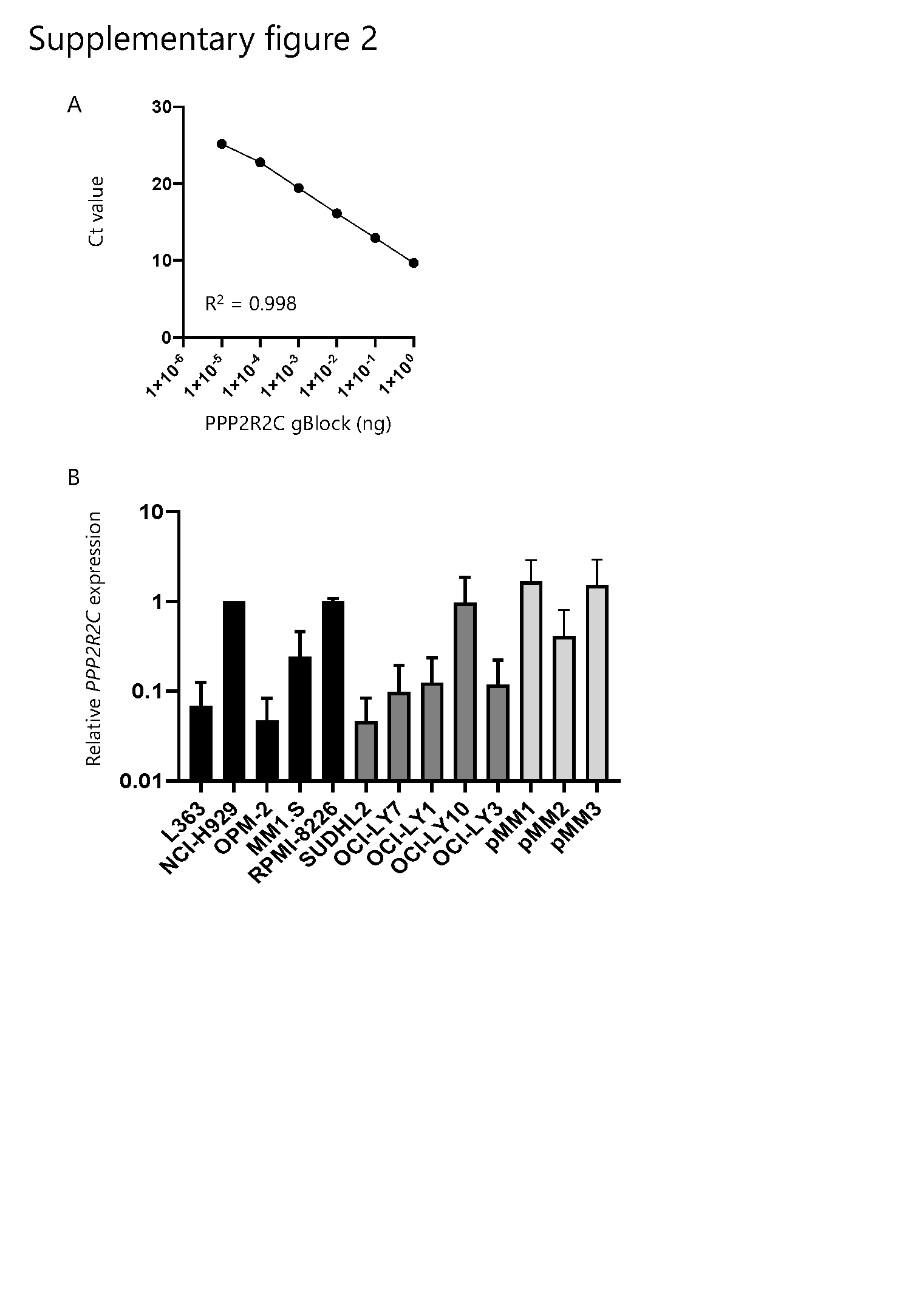

Supplement: Supplementary file 3 — Supplementary Figure 2 [file 41419_2020_3351_MOESM3_ESM.tif]

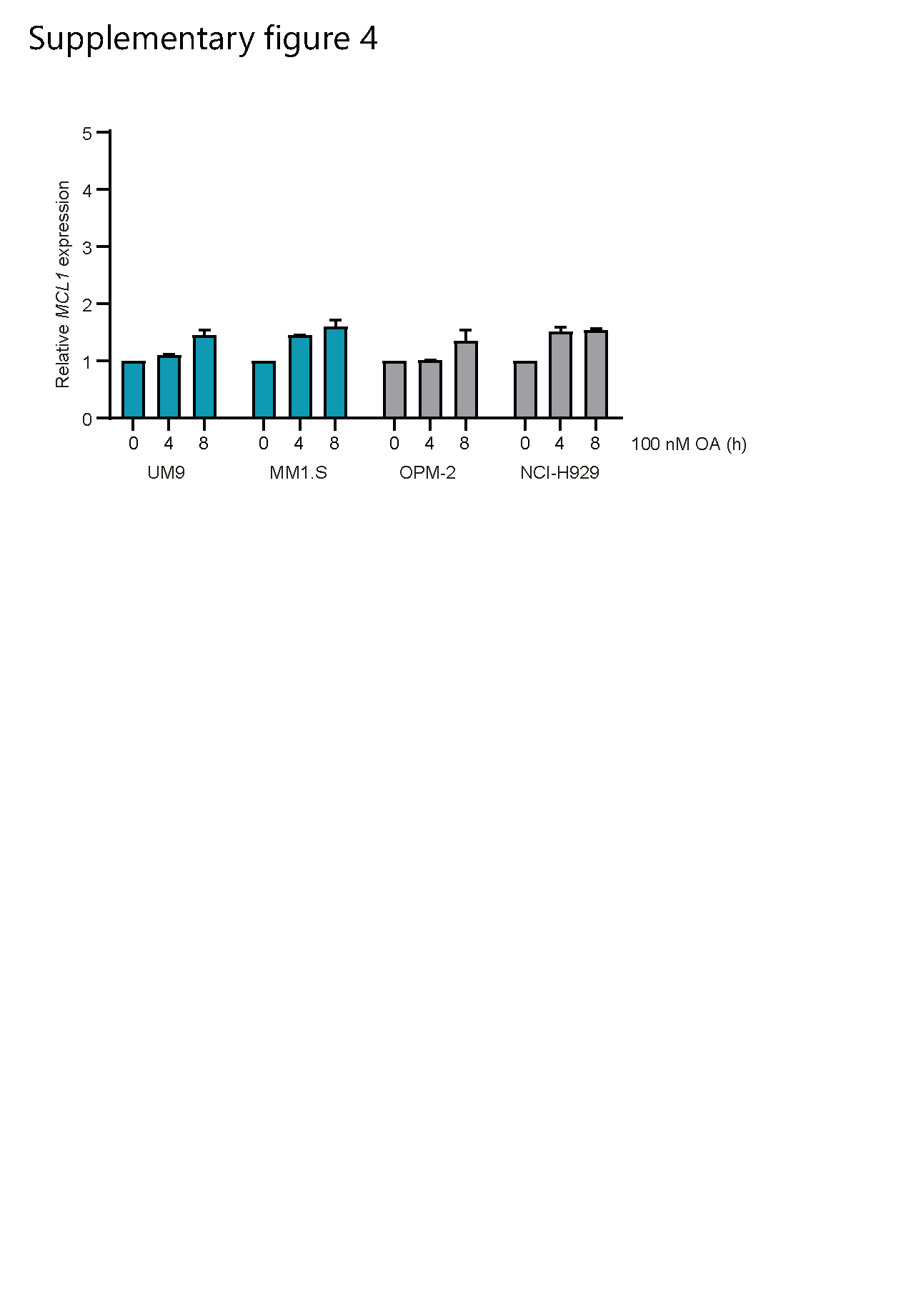

Supplement: Supplementary file 5 — Supplementary Figure 4 [file 41419_2020_3351_MOESM5_ESM.tif]
